# Gastric Mucosa-Associated Microbial Signatures of Early Gastric Cancer

**DOI:** 10.3389/fmicb.2020.01548

**Published:** 2020-07-07

**Authors:** Lili Wang, Yongning Xin, Jianhua Zhou, Zibin Tian, Chenguang Liu, Xinjuan Yu, Xinying Meng, Weina Jiang, Shoufeng Zhao, Quanjiang Dong

**Affiliations:** ^1^Central Laboratories, Department of Gastroenterology, Qingdao Municipal Hospital, Qingdao University, Qingdao, China; ^2^Department of Gastroenterology, The Affiliated Hospital of Qingdao University, Qingdao, China; ^3^College of Marine Life Sciences, Ocean University of China, Qingdao, China

**Keywords:** microbiome, gastric cancer, *Helicobacter pylori*, single nucleotide polymorphisms, *Ochrobactrum*

## Abstract

Alterations in the microbiome are associated with the development of gastric cancer. Our study aimed to identify dysbiotic features in early gastric cancer (EC). The gastric microbiome was assessed in EC (*n* = 30), advanced gastric cancer (AC) (*n* = 30), and chronic gastritis (CG) (*n* = 60). The results demonstrated significant differences in the microbial profile and composition between EC and AC, suggesting alterations associated with gastric cancer progression. Linear discriminant analysis (LDA) effect size (LEfSe) analyses identified 32 bacterial genera that were associated with EC. Functional analyses of the gastric microbiome showed that the production of urease and synthesis of bacterial flagella were weakened in EC, while the glycolysis of fructose and hydrolysis of glycosides were enhanced. A classifier based on a random forest (RF) machine learning algorithm identified a microbial signature that distinguished EC from CG or AC with high accuracy. The correct identification of the signature was further validated in independent cohorts. This signature enriched of bacteria with varied abundance, high degree of bacterial interactions and carcinogenic potentials. Constrained principal coordinate analyses revealed that the presence of *Helicobacter pylori* and the *cagA* and *vacA* virulence genotypes influenced the structure of the gastric microbiome. To determine the impacts of host genetic variations on the gastric microbiome, six previously reported single nucleotide polymorphisms (SNPs) were examined. The minor allele of *MUC1* rs4072037 was associated with an increased abundance of *Ochrobactrum*. The gastric microbiome altered in EC, which might be attributed in part to host genetic variations, *H. pylori* infection, bacterial virulence and environmental adaptations. The identified microbial signature could serve as biomarkers for clinical assessment of gastric cancer risk in high-risk patients.

## Introduction

Gastric cancer is a leading cause of cancer-related death (Lu and Li, 2014). Genetic variations and environmental factors such as *Helicobacter pylori* are involved in the development of gastric cancer. Recent studies suggest that the gastric microbiome is potentially involved in cancer development (Wang et al., 2014; Nardone et al., 2017). The development of *H. pylori*-induced gastric cancer is faster in mice containing artificial microflora in the stomach than in germ-free mice infected with the pathogen (Lofgren et al., 2011). During the carcinogenic process in the stomach, the gastric microbiome shows a continuous structural and compositional shift from mucosal inflammation to intestinal metaplasia and finally gastric cancer (Aviles-Jimenez et al., 2014). This finding suggests a close association between the microbiome and the development of gastric cancer. Analyses of the microbiome from two cities with contrasting incidences of gastric cancer reveal substantial differences in the microbial profile (Yang et al., 2016). Mice harboring different gastric microbiomes had differential incidences of inflammation and precancerous lesions (Ge et al., 2018). These findings indicate that variations in the microbiome contribute to carcinogenesis.

In gastric cancer, the microbiome of the stomach shows a distinct profile, altered biodiversity, enrichment and depletion of bacterial members, and changes in predicted functions (Wang et al., 2016; Castano-Rodriguez et al., 2017; Liu et al., 2019b). A distinct structure has been observed in the microbiome of gastric cancer that is different from that of chronic gastritis (CG) (Wang et al., 2016). Altered microbial biodiversity has been linked to the pathogenesis of many diseases. Increased species richness has been found in gastric cancer (Wang et al., 2016; Castano-Rodriguez et al., 2017; Liu et al., 2019b). Contrasting results regarding biodiversity have been reported (Ferreira et al., 2018), probably due to geographical and ethnic variations or variations in cancer stage. Oral bacteria and nitrosating bacteria are highly abundant in gastric cancer (Coker et al., 2018; Ferreira et al., 2018). Enrichment of oral bacteria has been linked to the occurrence of colorectal cancer. Levels of pathways related to carbohydrate metabolism and nitrate reductase are increased (Coker et al., 2018). Production of nitrite by bacteria promotes cancer development by increasing the levels of N-nitroso compounds (Wang et al., 2014).

Changes in the gastric microbiome in cancer are possibly carcinogenic. On the other hand, these changes merely reflect adaptive compositional changes in the gastric microbiome during cancer development. Host genetic variations, immunological status, infection and lowered acid output influence the composition of the gastric microbiome (Bonder et al., 2016; Li et al., 2017; Parsons et al., 2017). To date, few studies have been conducted to explore features of the microbial community in the early stage of gastric cancer. It is unclear whether dysbiotic features of the gastric microbiome could be useful in predicting early gastric cancer (EC). Development of gastric cancer is a multistep process from normal mucosa through mucosal inflammation and finally to cancer. In this study, we aimed to identify microbial signatures capable of classifying EC and to explore the influence of host genetic variations and *H. pylori* virulence on the composition of the gastric microbiome.

## Results

### Differences in Microbial Community Between Early and Advanced Gastric Cancer

**FIGURE 1:**
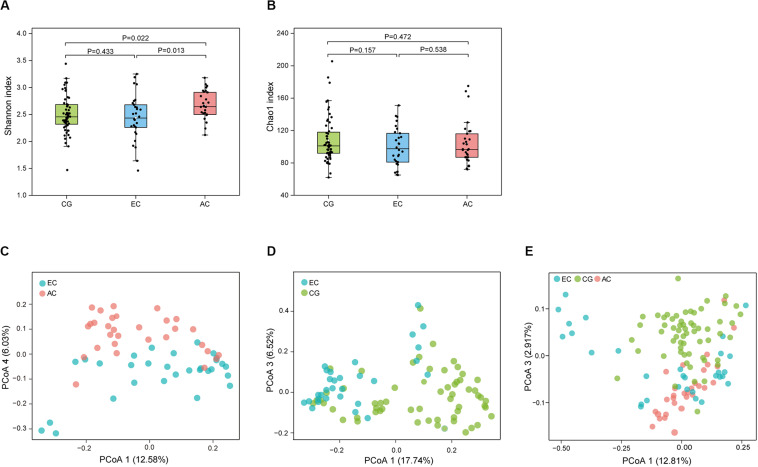
Microbial community profile among groups of gastric diseases. The alpha diversity of the gastric microbiome was estimated using the Shannon and Chao 1 indices. Dissimilarity in the structure of the gastric microbiome was assessed using Jaccard distance metrics. Box plot of the Shannon index **(A)** and Chao 1 index **(B)** in the CG, EC, and AC groups. PCoA of microbiome dissimilarity between EC and AC (**C**, *p* = 2.00E-04) or EC and CG (**D**, *p* = 1.00E-05). **(E)** PCoA analyses of the community structure between CG, EC, and AC (*p* values: EC vs. AC, 1.50E-03; EC vs. CG, 1.00E-05; AC vs. CG, 1.00E-05). CG, chronic gastritis; EC, early gastric cancer; AC, advanced gastric cancer.

**FIGURE 2:**
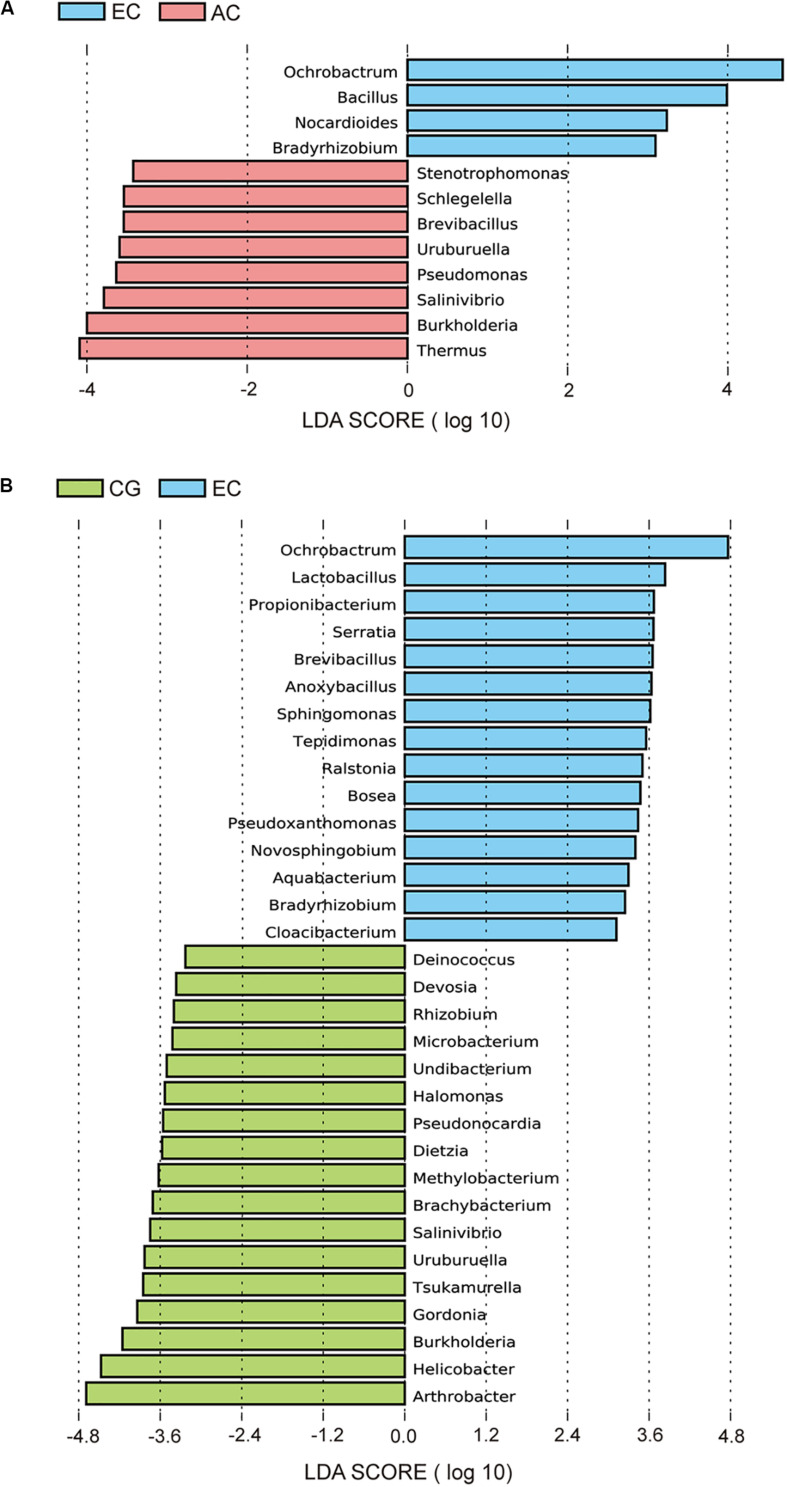
LEfSe analyses of microbiome composition between EC and AC **(A)** or between EC and CG **(B)**. Bacterial genera enriched in EC had a positive LDA score, while those depleted had a negative score. Bacteria with an LDA score greater than 2 are shown.

To determine the differences in the gastric microbiome between EC and advanced gastric cancer (AC), the alpha and beta diversities were measured. The Shannon index was significantly lower in EC than in AC (Figure 1A), demonstrating decreased biodiversity in EC. In contrast, there was no significant difference in the Chao 1 index between EC and AC (Figure 1B). To eliminate the possible influence of age, gender and *H. pylori* positivity on alpha diversity, linear regression analyses were performed for all samples (Supplementary Table S1). Age, gender and *H. pylori* positivity had no significant influence on the Shannon index (*p* = 0.398, 0.842, and 0.268, respectively) or the Chao 1 index (*p* = 0.126, 0.291, and 0.391, respectively). Dissimilarity in the structure of the gastric microbiome was measured using Jaccard distance matrices. Principal coordinate analysis (PCoA) showed apparent separation of the two stages of gastric cancer on the plot (Figure 1C). Therefore, the microbiome profile was distinct in the early stage of gastric cancer compared with the advanced stage of gastric cancer. Compositional analyses found no significant difference at the phylum level between the two stages of gastric cancer. *Proteobacteria* were predominant in both EC (0.546) and AC (0.547). At the genus level, linear discriminant analysis (LDA) effect size (LEfSe) analyses identified 12 genera with LDA scores higher than 2.0 (Figure 2A). A majority of these genera (8/12) were enriched in AC, suggesting a possible role in the progression of gastric cancer.

### Dysbiotic Gastric Microbiome in EC

Microbial profiles were assessed in EC and CG. In contrast to the significant difference in the biodiversity between EC and AC, the Shannon index showed no significant difference between EC and CG (Figure 1A). This finding suggested that biodiversity was not altered in EC. However, this index was significantly increased in AC compared with CG. PCoA analysis showed that the microbial community structure was discernable between EC and CG (Figure 1D). The structural differences of the gastric microbiome were also found between the three groups with PCoA analyses (Figure 1E). Thus, the community profile in EC was altered compared with that in CG. Analyses of the composition, however, revealed that 15 genera were more abundant in EC than in CG (Figure 2B). *Ochrobactrum* had the highest LDA score among these genera. *Helicobacter* and the other 16 genera showed decreased relative abundances in EC.

**FIGURE 3:**
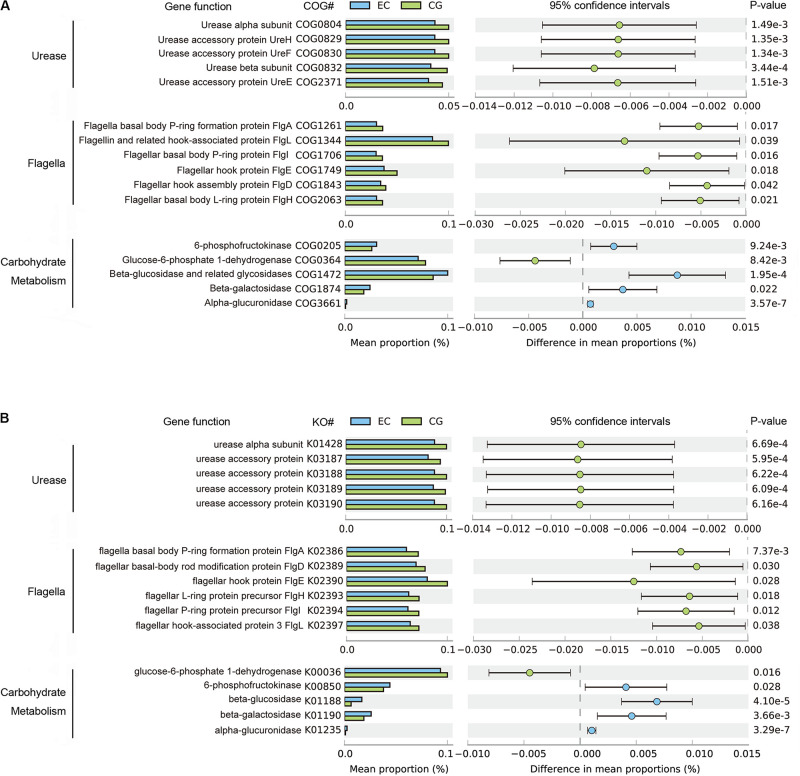
Differential functions predicted using PICRUSt between EC and CG. The mean relative frequency is shown for urease, flagella and carbohydrate metabolism, with significant differences in both COG **(A)** and KO **(B)** functional classifications. Differences between groups in the predicted functions were compared using STAMP. Statistical differences are considered for *p* < 0.05. COG, Clusters of Orthologous Groups; KO, Kyoto Encyclopedia of Genes and Genomes (KEGG) orthology.

The functional capacities of the gastric microbiome were predicted using PICRUSt. Compared with CG, the functional capacities were not significantly altered at Clusters of Orthologous Groups (COG) levels 1 and 2. However, there were considerable changes in pathways related to the biosynthesis of urease and flagella and to carbohydrate metabolism (Figure 3) at COG level 3. The average relative frequencies of pathways related to the structure and activity of urease and flagella were significantly decreased in EC, indicating reduced synthesis and activities of urease and motility (Figure 3A). These altered functional capacities were also found in the predicted Kyoto Encyclopedia of Genes and Genomes (KEGG) pathways (Figure 3B). Diverse pathways related to carbohydrate metabolism were altered. Increased relative frequency in EC was found for pathways related to glycolysis of fructose and hydrolysis of glycosides, including glucosides, galactosides, and saccharides. The relative frequency of glucose-6-phosphate dehydrogenase was decreased, suggesting that there was a reduction in carbohydrate degradation. These alterations were consistently found in both COG and KO functional analyses (Figure 3). In contrast, these altered functions showed no significant differences between EC and AC, except for 6-phosphofructokinase (COG205), the relative frequency of which decreased further in AC (Supplementary Table S2).

**FIGURE 4:**
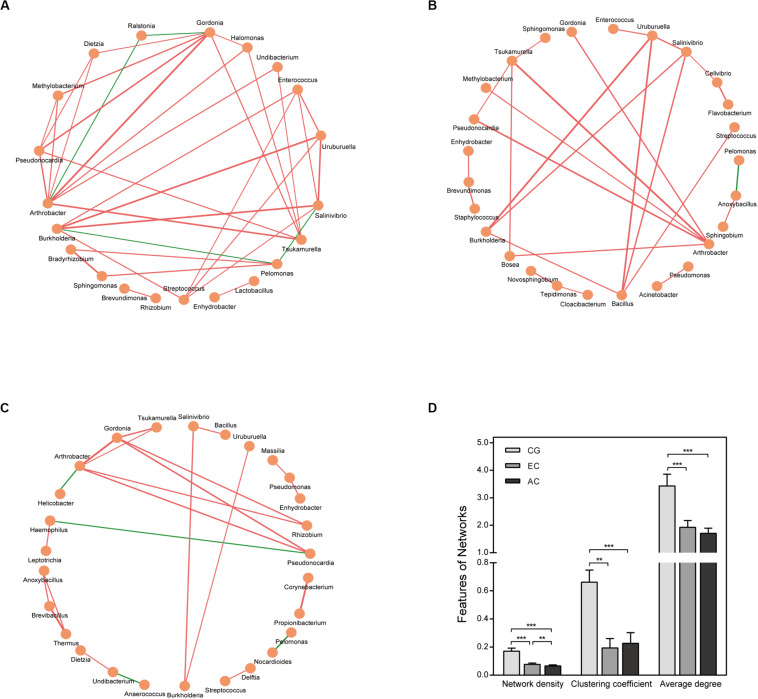
Correlation network of the gastric microbiome. The correlation coefficient was calculated with Spearman’s rank correlation test (| r| ≥ 0.6). Cytoscape version 3.7.1 was used for network construction. Correlation networks in CG **(A)**, EC **(B)**, and AC **(C)**; **(D)** Comparison of network features between CG and EC. Red and green lines represent positive and negative correlations, respectively. The thickness of the lines represents the size of the correlation. **p < 0.01; ***p < 0.001.

The correlation network differed greatly between CG and EC (Figure 4 and Supplementary Table S3). The network was larger in EC than in CG with an increased number of nodes. The network density and average degree, however, were lower in EC than in CG, demonstrating a reduction in the network complexity. The clustering coefficient of the network decreased, leading to an increased number of components and isolated sub-networks in EC. Therefore, the network in EC appeared to be fragmented. In AC, the network complexity appeared to be further reduced because the network density was lower than that in EC (Figures 4C,D).

### Microbial Signature Associated With EC

**FIGURE 5:**
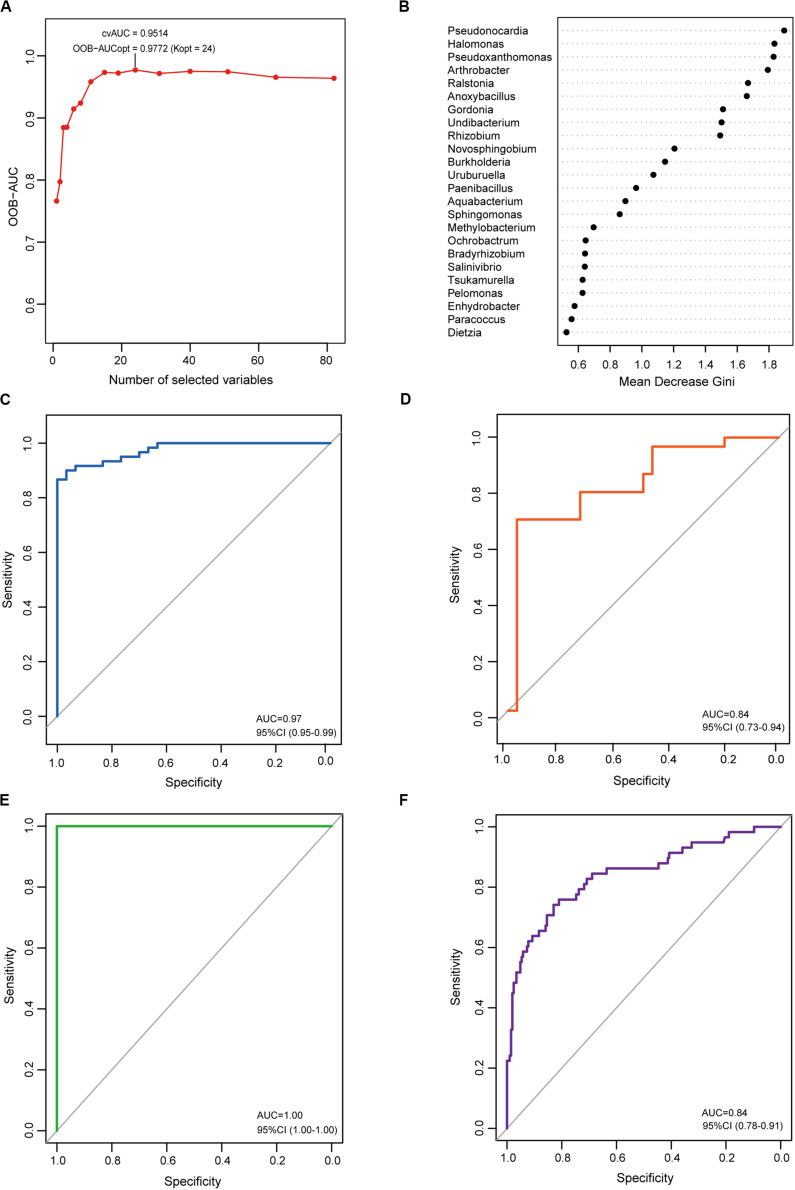
Identification and validation of the microbial signature associated with EC. To detect the optimal markers for EC, a RF model was constructed using the AUC-RF algorithm based on bacteria that were present in more than 20% samples and had a relative abundance over 0.05%. **(A)** The optimal model of 24 genera was selected by optimizing the area under the ROC curve of the random forest model. **(B)** The median decrease Gini (MDG) of selected genera in the optimal set. ROC curves of the optimal model for distinguishing EC from CG **(C)** and EC from AC **(D)**. To validate the identified markers, the trained model was used to predict gastric cancer in an independent Chinese cohort. ROC curves of the optimal model for distinguishing gastric cancer from healthy individuals **(E)** or from gastritis **(F)**.

To determine the microbial signature capable of discriminating EC from CG, a random forest (RF) classification model was built with the AUC-RF algorithm. The results showed a minimal set of 24 bacterial genera that maximally differentiated EC from CG (Figures 5A,B). RF models trained with this optimal set of features resulted in an out-of-bag error rate of 11.11%. To assess model classification accuracy, a 20-times repeated 10-fold cross-validation was performed. The area under curve (AUC) value from the cross-validation was 0.97 (95% CI: 0.95–0.99) (Figure 5C). The accuracy for distinguishing EC from CG appeared to be not influenced by the presence of intestinal metaplasia in CG (Supplementary Figures S1A,B). The trained model was further used to assess the accuracy for distinguishing EC from AC, and the results showed that this model was also capable of predicting EC with an AUC value of 0.84 (Figure 5D). In addition, the trained model could also distinguish AC from CG (Supplementary Figure S1C).

To explore the features of the bacteria in the optimal set, the relative abundance was analyzed. Compared with CG, most genera (12/24) were depleted in EC, and five genera (*Novosphingobium*, *Ochrobactrum*, *Ralstonia*, *Anoxybacillus*, and *Pseudoxanthomonas*) showed significantly increased abundances (Table 1). A majority of the genera (21/24) had LDA scores higher than 2.0. Compared with AC, only *Burkholderia*, *Tsukamurella*, *Uruburuella*, and *Salinivibrio* showed a decrease in the relative abundance, while the others had no significant change. Notably, the abundance of these four genera was also lower than that in CG. Results of LEfSe analyses found *Burkholderia*, *Uruburuella*, and *Salinivibrio*, in addition to *Ochrobactrum*, had an LDA score higher than 2.0. Analyses of the network revealed that these genera constituted a majority of the nodes in CG (17/21) and EC (13/26) and also comprised nearly half of the nodes in AC (13/27). These results suggested they were active in bacteria interaction and thus played dominant roles in the network.

**TABLE 1:** Features of the optimal set of bacterial genera identified with the random forest analysis.

Phylum	Genus	Average relative abundance (%)			*p*-value		LDA scores		Nodes in network		
		CG	EC	AC	EC vs. CG	EC vs. AC	EC vs. CG	EC vs. AC	CG	EC	AC
*Actinobacteria*	*Arthrobacter*	15.14 ± 6.76	7.85 ± 2.60	8.66 ± 3.24	1.67E-07	2.91E-01	4.69	NA	+	+	+
*Actinobacteria*	*Dietzia*	0.55 ± 0.56	0.28 ± 0.16	0.38 ± 0.29	1.11E-02	9.55E-02	3.57	NA	+	−	+
*Actinobacteria*	*Gordonia*	2.27 ± 1.09	1.09 ± 0.51	1.16 ± 0.54	1.99E-07	6.18E-01	3.94	NA	+	+	+
*Actinobacteria*	*Pseudonocardia*	0.82 ± 0.33	0.48 ± 0.25	0.53 ± 0.25	3.00E-06	4.72E-01	3.56	NA	+	+	+
*Actinobacteria*	*Tsukamurella*	2.92 ± 1.10	2.03 ± 0.75	2.59 ± 1.06	1.49E-04	2.27E-02	3.85	NA	+	+	+
*Firmicutes*	*Anoxybacillus*	0.10 ± 0.33	0.70 ± 1.19	0.66 ± 1.09	3.95E-04	8.78E-01	3.64	NA	−	+	+
*Firmicutes*	*Paenibacillus*	0.33 ± 0.52	0.31 ± 0.32	0.27 ± 0.27	8.45E-01	6.49E-01	NA	NA	−	−	−
*Proteobacteria*	*Aquabacterium*	0.16 ± 0.34	0.25 ± 0.18	0.29 ± 0.22	2.21E-01	3.93E-01	3.30	NA	−	−	−
*Proteobacteria*	*Bradyrhizobium*	0.25 ± 0.33	0.39 ± 0.32	0.27 ± 0.29	6.20 E-02	1.40E-01	3.25	3.10	+	−	−
*Proteobacteria*	*Burkholderia*	3.23 ± 3.08	1.35 ± 2.39	2.89 ± 1.76	4.32E-03	6.12E-03	4.16	4.00	+	+	+
*Proteobacteria*	*Enhydrobacter*	0.63 ± 0.67	1.80 ± 4.62	0.63 ± 0.58	5.51 E-02	1.72E-01	NA	NA	+	+	+
*Proteobacteria*	*Halomonas*	0.30 ± 0.17	0.13 ± 0.09	0.12 ± 0.11	3.02E-06	6.45E-01	3.53	NA	+	−	−
*Proteobacteria*	*Methylobacterium*	0.80 ± 0.42	0.43 ± 0.31	0.34 ± 0.23	5.89E-05	1.94E-01	3.62	NA	+	+	−
*Proteobacteria*	*Novosphingobium*	0.21 ± 0.29	0.53 ± 0.50	0.52 ± 0.40	2.27E-04	9.43E-01	3.40	NA	−	+	−
*Proteobacteria*	*Ochrobactrum*	20.81 ± 11.04	29.77 ± 11.47	24.27 ± 10.23	5.58E-04	5.48E-02	4.76	4.68	−	−	−
*Proteobacteria*	*Paracoccus*	0.20 ± 0.34	0.20 ± 0.30	0.14 ± 0.18	9.67 E-01	3.77E-01	NA	NA	−	−	−
*Proteobacteria*	*Pelomonas*	0.28 ± 0.44	0.28 ± 0.38	0.23 ± 0.32	9.85 E-01	5.98E-01	NA	NA	+	+	+
*Proteobacteria*	*Pseudoxanthomonas*	0.02 ± 0.03	0.23 ± 0.34	0.13 ± 0.29	6.34E-06	1.93E-01	3.44	NA	−	−	−
*Proteobacteria*	*Ralstonia*	0.06 ± 0.12	0.16 ± 0.16	0.18 ± 0.20	8.54E-04	6.17E-01	3.50	NA	+	−	−
*Proteobacteria*	*Rhizobium*	0.32 ± 0.21	0.15 ± 0.12	0.26 ± 0.33	7.22E-05	8.45E-02	3.40	NA	+	−	+
*Proteobacteria*	*Salinivibrio*	0.12 ± 0.17	0.03 ± 0.06	0.08 ± 0.09	6.09E-03	5.41E-03	3.75	3.79	+	+	+
*Proteobacteria*	*Sphingomonas*	1.74 ± 1.81	2.45 ± 1.48	2.37 ± 1.42	6.62 E-02	8.34E-01	3.62	NA	+	+	−
*Proteobacteria*	*Undibacterium*	0.16 ± 0.14	0.06 ± 0.06	0.06 ± 0.07	1.43E-04	6.31E-01	3.51	NA	+	+	+
*Proteobacteria*	*Uruburuella*	0.14 ± 0.16	0.04 ± 0.09	0.12 ± 0.12	2.18E-03	6.14E-03	3.83	3.59	+	+	+

*NA, LDA scores <2.00;+, presence in the correlation network; −, absence from the correlation network. CG, chronic gastritis; EC, early gastric cancer; AC, advanced gastric cancer.*

In order to evaluate whether the identified microbial signature could reflect the differences of the gastric microbiome between healthy individuals and gastric cancer, the RF analyses were performed on the dataset from Jiangxi, China available in Short Read Archive (SRA) of The National Center for Biotechnology Information (NCBI). It included raw data of the gastric microbiome from 28 healthy individuals, 206 patients with CG and 58 patients with gastric cancer. The results demonstrated the optimal set was capable of differentiating gastric cancer from healthy individuals with extremely high accuracy (Figure 5E). To further validate the identified microbial signature, classifying potential of the optimal set was evaluated on the datasets available in NCBI from populations of the above mentioned Jiangxi and Singapore. The optimal set identified differentiated Jiangxi patients with gastric cancer from those with CG with high accuracy (Figure 5F). The potential for classifying gastric cancer from functional dyspepsia was moderate in Singapore population (Supplementary Figure S2).

### Impacts of Virulent *H. pylori* Genotypes and Host Gene SNPs on the Gastric Microbiome

**FIGURE 6:**
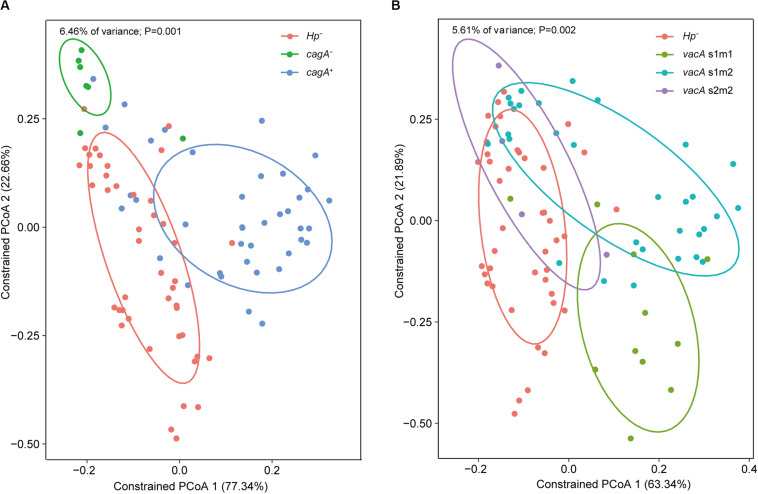
Impacts of *H. pylori* virulence on the gastric microbiome. Constrained PCoA were performed to explore the influence of *H. pylori* virulence on the gastric microbiome. Samples were separated between *cagA* genotypes **(A)** or between *vacA* genotypes **(B)** on the plots of the constrained PCoA.

Constrained PCoA were performed to explore the influence of *H. pylori* and its virulence on the gastric microbiome. First, genotyping was performed for the *vacA* and *cagA* genes, which are the major virulence determinants of *H. pylori*. Constrained PCoA demonstrated that *H. pylori* negative samples were clearly separated from *H. pylori* positive samples regardless of *cagA* positivity (Figure 6A), indicating that the presence of the pathogen had a significant impact on the structure of the gastric microbiome. This finding was supported by the result that samples lacking *H. pylori* were distinct from samples positive for the bacterium carrying the *vacA* s1m1 and s1m2 alleles (Figure 6B). Furthermore, samples with different *cagA* genotypes (*cagA*^+^ and *cagA*^–^) or *vacA* genotypes (s1m1, s1m2, and s2m2) were clearly separated from each other (Figures 6A,B). Therefore, *H. pylori* virulence impacted the composition of the gastric microbiome.

Gastric cancer-associated SNPs of the host were determined (Supplementary Table S4). Constrained PCoA was conducted to explore the influence of SNPs on the gastric microbiome. Samples of different SNP genotypes showed no clear separation, indicating that cancer-associated SNPs had little impact on the structure of the gastric microbiome (Supplementary Figure S3). Nonetheless, comparison of the relative abundance between SNP genotypes demonstrated an association of *Ochrobactrum* with *MUC1* SNP rs4072037. The relative abundance of *Ochrobactrum* was 0.28 ± 0.12 in minor allele C, which was significantly higher than that in allele T (0.23 ± 0.11, *p* = 0.016).

## Discussion

In this study, our results demonstrated significant differences between EC and AC in the biodiversity and structure of the gastric microbiome. This finding suggests that the microbiome is altered in the progression from the early stage to the advanced stage of gastric cancer. Furthermore, our study found a total of 12 bacteria had a LDA value greater than 2.0, suggesting an association between these bacteria genera and the progression of gastric cancer. The gastric microbiome has been poorly characterized in the progression of gastric cancer. Our study demonstrate substantial variations of the gastric microbiome in the progression of gastric cancer, consisting with the findings that the microbial community show remarkable differences between early and advanced colorectal cancer (Xie et al., 2017; Nakatsu et al., 2018).

Altered microbial biodiversity is a typical feature of dysbiosis and has been linked to various diseases (Gong et al., 2016). Our findings demonstrated an increased diversity in advanced-stage gastric cancer, which is consistent with other studies on gastric cancer (Wang et al., 2016; Ferreira et al., 2018). However, no significant alteration in biodiversity was found in the early stage of cancer. Therefore, the observed changes in biodiversity and structure in gastric cancer most likely occur during cancer progression rather than during the carcinogenic process. In contrast to our findings, a decrease in alpha diversity has been reported in gastric cancer (Aviles-Jimenez et al., 2014; Coker et al., 2018; Ferreira et al., 2018). Differences in populations studied, sample size and other factors, including smoking status, co-medications and dietary habits, might contribute to the conflicting alpha-diversity results. Further studies are required to clarify this issue. Previous reports have found that many bacteria are enriched or depleted in gastric cancer. However, it is unclear regarding the compositional changes of the gastric microbiome in EC. Our study found 32 bacterial genera were associated with EC. Their potential role in the carcinogenesis needs to be explored in the future.

Microbial signatures have been recently identified to discriminate between different diseases. These signatures can be used as biomarkers for early detection of gastrointestinal cancer (Mangifesta et al., 2018; Xu et al., 2019). A few studies have been conducted to identify microbial signatures in gastric cancer. In a recent report, the microbial dysbiosis index was used to predict gastric cancer (Ferreira et al., 2018). This index was calculated based on 10 relevant bacteria with differential abundances between gastric cancer and CG. In another report, a combination of five bacteria, which were highly interactive and enriched in GC, was used to classify gastric cancer and non-cancer samples (Coker et al., 2018). Both studies showed that the identified signature could predict cancer with high accuracy. Despite this finding, the microbial signature has not yet been explored in the early stage of gastric cancer. In this study, the microbial signature of EC was identified using the RF machine learning algorithm. The trained model consisted of a set of 24 bacterial genera capable of predicting early stage cancer as well as advanced stage cancer with considerably high accuracy. Moreover, the capacity of the trained model for distinguishing EC from CG appeared to be not influenced by the presence of intestinal metaplasia in CG. The identified signatures were further validated in independent populations. We found the signatures classified gastric cancer from healthy controls with a AUC value equal to 1.0, validating the correct identification of the microbial signatures. Our findings also revealed the high capacity of the identified signatures for differentiating gastric cancer from gastritis in China and Singapore populations, further validating the classifying potential of the signatures. Nonetheless, the potential appeared to be lower in populations other Chinese, which might be attributed to geographical variations of the gastric microbiome. These results demonstrated the identified microbial signature is highly capable of discriminating EC from normal gastric mucosa, CG and AC. Thus, the signature would potentially be biomarkers for early detection of gastric cancer.

Our study found most bacteria enclosed in the identified signature had differential abundance between EC and CG groups. Results of LEfSe analyses also demonstrated they had high LDA values. In addition, the network analyses revealed the majority of these bacteria interacted with other bacteria of the gastric microbiome. These suggest the microbial signature consists of a set of bacteria featuring with varied abundance and a high degree of bacterial interactions during the development of gastric cancer. Furthermore, certain of them may possess carcinogenic potentials. *Novosphingobium*, *Ralstonia*, *Ochrobactrum*, *Anoxybacillus*, and *Pseudoxanthomonas* were enriched in EC. Previous studies have found *Novosphingobium*, *Anoxybacillus*, and *Ralstonia* are associated with gastric cancer, extrahepatic cholangiocarcinoma and bladder cancer (Avilés-Jiménez et al., 2016; Tseng et al., 2016; Liu et al., 2019a). They play a role in the initiation of inflammation (Rutebemberwa et al., 2014; Tejera et al., 2016). Several species of *Ochrobactrum* have been isolated from the human stomach (Dharne et al., 2008; Kulkarni et al., 2014). It has been suggested that they are pathogens causing gastric diseases (Kulkarni et al., 2014). Our study found only *Burkholderia*, *Tsukamurella*, *Uruburuella*, and *Salinivibrio* of the signature showed variation in the abundance between EC and AC. Their abundance was lower than that of CG or AC. Depletion of certain bacteria has been associated with cancer (Hayes et al., 2018). The association between the depletion of these four bacteria and carcinogenesis remains to be clarified.

Cancer-associated microbiome shows substantial changes in the functional profile (Yang and Jobin, 2017). A prominent and consistent functional change in the gastric cancer-associated microbiome is the change in the enrichment pathways related to carbohydrate metabolism (Castano-Rodriguez et al., 2017; Ferreira et al., 2018). Consistent with this finding, our results demonstrated alterations in the pathways related to glycolysis of fructose and hydrolysis of glycosides in EC. This result may reflect compositional changes in the microbiome. Nonetheless, it has been suggested that metabolites of carbohydrate metabolism possibly contribute to cell hyperproliferation and carcinogenesis (Belcheva et al., 2014). Furthermore, we found weakened motility and decreased urease activity in EC. Both motility and urease activity play important roles in bacterial survival and proliferation in acidic environments (Guo and Mekalanos, 2002; Sachs et al., 2003). In patients with gastric cancer, the acid output of the stomach is decreased. Thus, urease activity and motility no longer provide a growth advantage, leading to growth inhibition of urease-containing bacteria and/or motile bacteria. Therefore, altered functions most likely reflect adaptive compositional changes in the microbiome in response to altered environments. In the gastric microbiome, *H. pylori* is the major contributor of urease. The observed reduction in urease activity is possibly caused by the decreased quantity of *H. pylori*, although the positivity rate of the pathogen was similar between CG and EC in our study. Consistent with this finding, it has been shown that *H. pylori* may gradually disappear during the progression from the inflamed gastric mucosa to cancer due to pathological changes in the mucosa. Our results found that there were no significant differences in these predicted functions between EC and AC, suggesting that the functional changes in the gastric microbiome remained during cancer progression.

Bacterial interaction is a determinant of microbiome homeostasis. The network complexity is reduced in peritumoral or tumoral tissues of the stomach in comparison with normal gastric mucosa (Liu et al., 2019b). A recent report, however, argued that the network complexity was increased in gastric cancer (Ferreira et al., 2018). Our results demonstrated that the microbial network was simplified in the early stage of gastric cancer as well as AC. Furthermore, the network was fragmented. These findings suggest the loss of certain bacterial interactions in gastric cancer, leading to disruption of the homeostasis of the gastric microbiome. Our results also showed the network indices including network density, clustering coefficient and average degree was significantly different between CG and gastric cancer. Whether they could be used as quantitative parameters to assess cancer risk and homeostasis of the gastric microbiome requires further study.

*Helicobacter pylori* is a pathogen that colonizes the human stomach. Infection by this pathogen leads to altered structure of the gastric microbiome (Wang et al., 2016; Ren et al., 2018; Gantuya et al., 2019). In consistence with this finding, our data demonstrated impacts of *H. pylori* on the structure of the gastric microbiome. Furthermore, our findings showed that virulent genotypes of *H. pylori* are associated with variations in the microbial profile. This finding indicates that bacterial virulence may influence the composition of the gastric microbiome. Further studies are required to clarify the contribution of *H. pylori* virulence to the dysbiosis of the microbiota during gastric carcinogenesis. In this study, we found that cancer-associated SNPs were not correlated with variations in microbial profiles. This fact is consistent with findings from studies on the gut microbiome that host genetic variations generally do not alter the structures of microbial communities (Murphy et al., 2015; Goodrich et al., 2016). Our results, however, demonstrated that the *MUC1* SNP rs4072037 was associated with *Ochrobactrum*. This abundance of this SNP increased in the carcinogenic minor allele of rs4072037. *Ochrobactrum* is a stomach pathogen. Therefore, it seems possible that *MUC1* SNP rs4072037 could contribute to cancer development by influencing the gastric composition. The cell surface mucin MUC1 is a large glycoprotein which is highly expressed in the surface of gastric mucosa. Despite of functioning as a receptor for bacterial adhesins, it could limits the density of *H. pylori* in a murine infection model (Mcguckin et al., 2007). MUC1 may inhibit adhesion of non-MUC1 binding bacteria to the gastric epithelium (Linden et al., 2009), reducing the density of the bacteria. For MUC1-binding bacteria, it acts as a releasable decoy to decrease the density of bacteria (Linden et al., 2009). Therefore, deficiency in MUC1 may increase the abundance of the microbial members in the gastric microbiome. Further demonstration of the functional association between *MUC1* SNP rs4072037 with the density of *Ochrobactrum* is indicated.

## Conclusion

In summary, this study revealed alterations of the gastric microbiome in the early stage of gastric cancer featured adaptive functional and compositional changes and a simplified network. Host genetic backgrounds, *H. pylori* virulence and environmental changes may contribute to the alterations of the gastric microbiome during the development and progression of gastric cancer. We identified a microbial signature that was capable of accurately distinguishing EC from CG or AC. The signature showed characteristics of varying abundance, high degree of bacteria interaction and carcinogenic potentials. It could serve as biomarkers for clinical assessment of gastric cancer risk in high-risk patients.

## Materials and Methods

### Patients and Sample Collection

A total of 120 patients were enrolled in the study between January 2015 and November 2018. These patients underwent endoscopic examination at Qingdao Municipal Hospital due to complaints of upper gastrointestinal symptoms. Of these patients, 85 were male. The mean age of the patients was 55.9 ± 12.1 years. All patients were of Chinese Han ethnicity. These patients included 60 patients with CG, 30 with EC, and 30 with AC. There were no significant differences in gender between groups (Supplementary Table S1). However, the average age was lower in CG than in the other groups. For patients with CG, endoscopy findings showed only the appearance of CG without any other lesions (ulcer, polyp or bleeding). Data for the degree and activity of mucosal inflammation in CG patients is shown in Supplementary Table S1. Histologically, 30 of the patients with CG showed evidence of intestinal metaplasia. None of the 60 patients with CG had advanced gastric atrophy or dysplasia. For patients with gastric cancer, only non-cardia gastric adenocarcinoma was included. Pathological staging was conducted based on the surgically removed stomach (Supplementary Table S1). EC was defined as a tumor with invasion limited to the mucosa or submucosa of the stomach, irrespective of lymph node involvement (Pimentel-Nunes et al., 2015). Tumors infiltrating beyond the submucosal layer of the stomach were defined as AC (Fugazzola et al., 2018). The *H. pylori* status was determined pathologically using a modified Giemsa staining method as previously reported (Wang et al., 2016). There were no significant differences in the positive rate of *H. pylori* between the groups (Supplementary Table S1). All of the enrolled subjects had no history of diabetes mellitus or any other severe complications, including heart, liver, and renal failure. None of the patients had received any antibiotics or proton pump inhibitor treatment 8 weeks prior to the examination. Antral biopsies were taken during endoscopic examinations. For patients with gastric cancer, a biopsy was taken at least 5 cm away from the cancerous lesion. Biopsies were stored at −80°C until use.

### Analyses of the Gastric Microbiome

To analyze the microbial communities of the gastric mucosa, genomic DNA was extracted from gastric mucosa samples as previously reported (Wang et al., 2016). The variable V3–V4 region of the 16S rRNA gene was PCR amplified with primers 338F/806R to generate the amplicon libraries. Sequencing was performed on a HiSeq 2500 platform (Illumina, Hayward, CA, United States). A total of 19,656,799 paired-ends reads were obtained. After quality control and filtration, 16,333,787 reads were produced with an average of 136,115 reads per sample. The sequence datasets have been submitted to Sequence Read Archive (SRA) of NCBI^1^. The BioProject accession number is PRJNA313391. The reads were analyzed using UPARSE (Edgar, 2013). Following global trimming at 250 nucleotides, reads were dereplicated, and singletons were discarded. Subsequently, reads were clustered into operational taxonomic units (OTUs) assuming 97% identity. Chimeric reads were then removed. Taxonomy assignation was performed using UClust (Edgar, 2010). Analyses of alpha and beta microbial diversity were conducted as described previously (Wang et al., 2016). Comparisons of the relative abundances of taxa between groups were performed using version 1.0 of LEfSe (Segata et al., 2011). An LDA value greater than 2 at a *p* value less than 0.05 was considered statistically significant. To analyze the correlation network, Spearman correlations were computed between the genera in different groups. The correlations that had an absolute Spearman coefficient values greater than or equal to 0.6 with a *p* value greater than 0.05 were transformed into links between two genera in the genus network. Cytoscape v3.7.1 was then used to construct network figures. For predicting the functions of the microbial community, PICRUSt (v1.1.1) was used (Langille et al., 2013). The accuracy of the predicted metagenomes was assessed by the nearest sequenced taxon index (NSTI). Predicted functions were categorized with COG and KEGG orthology. STAMP (v2.1.3) was used to compare patient groups (Parks et al., 2014).

In addition, data of the gastric microbiome was also downloaded from SRA databases, NCBI and re-analyzed following the aforementioned methods. These included a Chinese cohort from Jiangxi consisting of 445 samples (Bioproject accession number: PRJNA481413). A total of 292 samples, including 8 healthy controls, 206 samples from CG and 58 samples from gastric cancer, was used for re-analyzing the gastric microbiome. The other samples including 96 gastric fluid samples and 57 cancerous samples were excluded from further analyses. A Singapore cohort consisting of 36 samples (23 from Malaysia and 13 from Singapore) was also downloaded (Bioproject accession number: PRJEB21497). Samples of this cohort were from 20 patients with functional dyspepsia and 12 with gastric cancer. A total of four samples from peptic ulcer diseases were not included for further analyses.

### Genotyping of SNPs

Based on findings from previous genome-wide association studies, a total of six SNPs associated with high risk for gastric cancer were selected for analyses (Shi et al., 2011; Helgason et al., 2015; Hu et al., 2016). These SNPs included rs2920299 and rs2976392 in *PSCA*, rs2294693 in *UNC5CL*, rs80315667 in *PRKAA1*, rs10036575 in *PTGER*, and rs4072037 in *MUC1* genes. To determine the genotypes of these SNPs, genomic DNA was extracted from venous blood samples. Genotyping was performed with the Sequenom MassArray system (San Diego, CA, United States) essentially according to the manufacturer’s instructions. Primers were designed using MassArray Assay Design 4.0 (Sequenom). The resultant mass spectrograms and genotype data were analyzed using MassArray Typer 4.0 software.

### Determination of Virulent Genotypes of *H. pylori*

To determine virulent genotypes of *H. pylori* directly from gastric mucosa samples, PCR amplifications of *vacA* and *cagA* were performed according to previous reports (Atherton et al., 1995, 1999).

### Statistical Analysis

To identify microbial signatures capable of distinguishing EC or AC from CG, a RF model was built using the AUC-RF algorithm (Calle et al., 2011). The input variables comprised the relative abundances of taxa and/or the genotyping results. A taxon was included only if it was present in more than 20% samples and had a relative abundance over 0.05%. A 20-times repeated 10-fold cross-validation of the RF model was performed. The performance of the RF model was demonstrated by the receiver operating characteristic (ROC) curve (Hanley and Mcneil, 1982).

The Mann–Whitney *U* test was performed to detect significant differences in alpha diversity between disease groups or relative abundances between groups. Constrained PCoA was performed in R to explore the influence of cancer-associated SNPs and the status and virulent genotypes of *H. pylori* on the gastric microbiome (Anderson and Willis, 2003).

## Data Availability Statement

The datasets presented in this study can be found in online repositories. The names of the repository/repositories and accession number(s) can be found in the article/Supplementary Material.

## Ethics Statement

The studies involving human participants were reviewed and approved by the Research Ethnics Committee of Qingdao Municipal Hospital, China. The patients/participants provided their written informed consent to participate in this study.

## Author Contributions

QD and YX contributed to the design of the study. LW and JZ wrote the original draft of the manuscript and performed the experiments. QD, ZT, and CL reviewed and edited the manuscript. XY and XM contributed to sample and clinical data collection. YX and WJ performed the bioinformatics and statistical analysis. XM and SZ contributed to the validation. All authors critically reviewed the manuscript and approved the final version of the manuscript.

## Conflict of Interest

The authors declare that the research was conducted in the absence of any commercial or financial relationships that could be construed as a potential conflict of interest.

## Footnotes

1 https://www.ncbi.nlm.nih.gov/bioproject/?term=PRJNA313391
