# Supplementary material for: Gastric Mucosa-Associated Microbial Signatures of Early Gastric Cancer
Source: Front Microbiol. 2020 Jul 7;11:1548. doi: 10.3389/fmicb.2020.01548 (PMC7358557; doi:10.3389/fmicb.2020.01548)
Supplement: Supplementary file 1 [file Table_1.DOCX]

**Supplementary Table S1 Clinical and pathological characteristics of the patients.**

| Characteristics | CG  (n=60) | EC  (n=30) | AC  (n=30) | *p*-value | | |
| --- | --- | --- | --- | --- | --- | --- |
|  |  |  |  | **EC vs. CG** | **EC vs. AC** | **AC vs. CG** |
| Age (years) | 51.58±11.06 | 60.07±11.52 | 60.27±11.98 | 0.001 | 0.946 | 0.001 |
| Sex (M/F) | 41/19 | 23/7 | 21/9 | 0.411 | 0.559 | 0.872 |
| *H. pylori* positivity (%) | 32 (53.3) | 14 (46.7) | 15 (50.0) | 0.551 | 0.796 | 0.765 |
| TNM classification |  |  |  |  |  |  |
| T1N0M0 |  | 27 |  |  |  |  |
| T1N1M0 |  | 2 |  |  |  |  |
| T1N2M0 |  | 1 |  |  |  |  |
| T2N0M0 |  |  | 4 |  |  |  |
| T2N1M0 |  |  | 1 |  |  |  |
| T2N3M0 |  |  | 1 |  |  |  |
| T4N0M0 |  |  | 6 |  |  |  |
| T4N1M0 |  |  | 3 |  |  |  |
| T4N2M0 |  |  | 3 |  |  |  |
| T4N3M0 |  |  | 12 |  |  |  |
| Degree of inflammation |  |  |  |  |  |  |
| Mild | 7 |  |  |  |  |  |
| Moderate | 21 |  |  |  |  |  |
| Severe | 32 |  |  |  |  |  |
| Activity of inflammation |  |  |  |  |  |  |
| None | 4 |  |  |  |  |  |
| Mild | 31 |  |  |  |  |  |
| Moderate | 20 |  |  |  |  |  |
| Severe | 5 |  |  |  |  |  |

CG, chronic gastritis; EC, early gastric cancer; AC, advanced gastric cancer.
