# Supplementary material for: Gastric Mucosa-Associated Microbial Signatures of Early Gastric Cancer
Source: Front Microbiol. 2020 Jul 7;11:1548. doi: 10.3389/fmicb.2020.01548 (PMC7358557; doi:10.3389/fmicb.2020.01548)
Supplement: Supplementary file 2 [file Table_2.DOCX]

**Supplementary Table S2 Differences between EC and AC in functions of the gastric microbiome predicted using PICRUSt.**

| **Function** | **COG/KO** | **AC: mean rel. freq. (%)** | **AC: std. dev. (%)** | **EC: mean rel. freq. (%)** | **EC: std. dev. (%)** | ***p*-values (corrected)** | **Difference between means** | **95.0% lower CI** | **95.0% upper CI** |
| --- | --- | --- | --- | --- | --- | --- | --- | --- | --- |
| Urea | COG0804 | 0.0403 | 0.0052 | 0.0428 | 0.0096 | 0.2209 | -0.0025 | -0.0066 | 0.0015 |
| Urea | COG0829 | 0.0402 | 0.0052 | 0.0427 | 0.0096 | 0.2166 | -0.0025 | -0.0066 | 0.0015 |
| Urea | COG0830 | 0.0402 | 0.0052 | 0.0427 | 0.0096 | 0.2165 | -0.0025 | -0.0066 | 0.0015 |
| Urea | COG0832 | 0.0393 | 0.0051 | 0.0408 | 0.0107 | 0.5174 | -0.0014 | -0.0058 | 0.0030 |
| Urea | COG2371 | 0.0370 | 0.0048 | 0.0397 | 0.0096 | 0.1901 | -0.0026 | -0.0066 | 0.0013 |
| Flagella | COG1261 | 0.0259 | 0.0042 | 0.0263 | 0.0082 | 0.8239 | -0.0004 | -0.0038 | 0.0030 |
| Flagella | COG1344 | 0.0708 | 0.0120 | 0.0737 | 0.0218 | 0.5315 | -0.0029 | -0.0122 | 0.0063 |
| Flagella | COG1706 | 0.0256 | 0.0041 | 0.0260 | 0.0082 | 0.7910 | -0.0005 | -0.0038 | 0.0029 |
| Flagella | COG1749 | 0.0322 | 0.0081 | 0.0327 | 0.0163 | 0.8747 | -0.0005 | -0.0073 | 0.0062 |
| Flagella | COG1843 | 0.0287 | 0.0039 | 0.0299 | 0.0072 | 0.4263 | -0.0012 | -0.0043 | 0.0018 |
| Flagella | COG2063 | 0.0258 | 0.0040 | 0.0264 | 0.0082 | 0.7546 | -0.0005 | -0.0039 | 0.0029 |
| Carbohydrate Metabolism | COG0205 | 0.0218 | 0.0036 | 0.0190 | 0.0050 | 0.0217 | 0.0027 | 0.0004 | 0.0050 |
| Carbohydrate Metabolism | COG0364 | 0.0448 | 0.0021 | 0.0439 | 0.0074 | 0.5326 | 0.0009 | -0.0020 | 0.0038 |
| Carbohydrate Metabolism | COG1472 | 0.0597 | 0.0047 | 0.0616 | 0.0111 | 0.4052 | -0.0019 | -0.0064 | 0.0026 |
| Carbohydrate Metabolism | COG1874 | 0.0138 | 0.0029 | 0.0151 | 0.0112 | 0.5505 | -0.0013 | -0.0056 | 0.0030 |
| Carbohydrate Metabolism | COG3653 | 0.0094 | 0.0018 | 0.0096 | 0.0046 | 0.8559 | -0.0002 | -0.0020 | 0.0017 |
| Carbohydrate Metabolism | COG3661 | 0.0010 | 0.0006 | 0.0011 | 0.0007 | 0.2807 | -0.0002 | -0.0005 | 0.0002 |
| Urea | K01428 | 0.0592 | 0.0079 | 0.0627 | 0.0128 | 0.2051 | -0.0036 | -0.0092 | 0.0020 |
| Urea | K03187 | 0.0545 | 0.0072 | 0.0582 | 0.0127 | 0.1755 | -0.0037 | -0.0091 | 0.0017 |
| Urea | K03188 | 0.0591 | 0.0079 | 0.0627 | 0.0128 | 0.2044 | -0.0036 | -0.0092 | 0.0020 |
| Urea | K03189 | 0.0584 | 0.0077 | 0.0619 | 0.0127 | 0.2073 | -0.0035 | -0.0091 | 0.0020 |
| Urea | K03190 | 0.0591 | 0.0079 | 0.0627 | 0.0128 | 0.2053 | -0.0036 | -0.0092 | 0.0020 |
| Flagella | K02386 | 0.0368 | 0.0055 | 0.0372 | 0.0107 | 0.8465 | -0.0004 | -0.0049 | 0.0040 |
| Flagella | K02389 | 0.0414 | 0.0053 | 0.0432 | 0.0091 | 0.3718 | -0.0018 | -0.0057 | 0.0022 |
| Flagella | K02390 | 0.0488 | 0.0106 | 0.0502 | 0.0195 | 0.7395 | -0.0014 | -0.0096 | 0.0069 |
| Flagella | K02393 | 0.0379 | 0.0053 | 0.0387 | 0.0106 | 0.7364 | -0.0007 | -0.0052 | 0.0037 |
| Flagella | K02394 | 0.0375 | 0.0054 | 0.0381 | 0.0106 | 0.7755 | -0.0006 | -0.0050 | 0.0038 |
| Flagella | K02397 | 0.0380 | 0.0052 | 0.0396 | 0.0091 | 0.4275 | -0.0016 | -0.0054 | 0.0023 |
| Carbohydrate Metabolism | K00036 | 0.0620 | 0.0025 | 0.0610 | 0.0098 | 0.6134 | 0.0010 | -0.0028 | 0.0047 |
| Carbohydrate Metabolism | K00850 | 0.0334 | 0.0051 | 0.0289 | 0.0079 | 0.0125 | 0.0045 | 0.0010 | 0.0080 |
| Carbohydrate Metabolism | K01188 | 0.0085 | 0.0036 | 0.0110 | 0.0116 | 0.2684 | -0.0025 | -0.0070 | 0.0020 |
| Carbohydrate Metabolism | K01190 | 0.0213 | 0.0113 | 0.0169 | 0.0067 | 0.0769 | 0.0044 | -0.0005 | 0.0093 |
| Carbohydrate Metabolism | K01235 | 0.0013 | 0.0009 | 0.0016 | 0.0010 | 0.2125 | -0.0003 | -0.0008 | 0.0002 |
| Carbohydrate Metabolism | K01461 | 0.0002 | 0.0001 | 0.0003 | 0.0002 | 0.0072 | -0.0001 | -0.0002 | 0.0000 |
