# Supplementary material for: Gastric Mucosa-Associated Microbial Signatures of Early Gastric Cancer
Source: Front Microbiol. 2020 Jul 7;11:1548. doi: 10.3389/fmicb.2020.01548 (PMC7358557; doi:10.3389/fmicb.2020.01548)
Supplement: Supplementary file 3 [file Table_3.DOCX]

**Supplementary Table S3 Spearman correlation between genera in different diseases.**

| **Spearman correlation between genera in CG** | | | | | |
| --- | --- | --- | --- | --- | --- |
| **Source** | **Target** | | **Spearman r value** | | ***p*-value** |
| *Arthrobacter* | *Gordonia* | | 0.90 | | 7.84E-23 |
| *Burkholderia* | *Uruburuella* | | 0.86 | | 5.53E-19 |
| *Burkholderia* | *Salinivibrio* | | 0.86 | | 1.41E-18 |
| *Uruburuella* | *Salinivibrio* | | 0.84 | | 5.34E-17 |
| *Arthrobacter* | *Tsukamurella* | | 0.83 | | 1.72E-16 |
| *Sphingomonas* | *Bradyrhizobium* | | 0.82 | | 5.44E-16 |
| *Pseudonocardia* | *Gordonia* | | 0.80 | | 9.40E-15 |
| *Pseudonocardia* | *Arthrobacter* | | 0.79 | | 7.94E-14 |
| *Methylobacterium* | *Gordonia* | | 0.75 | | 5.67E-12 |
| *Sphingomonas* | *Pelomonas* | | 0.73 | | 2.36E-11 |
| *Enterococcus* | *Uruburuella* | | 0.71 | | 1.94E-10 |
| *Pseudonocardia* | *Tsukamurella* | | 0.71 | | 3.11E-10 |
| *Burkholderia* | *Enterococcus* | | 0.70 | | 5.47E-10 |
| *Pelomonas* | *Bradyrhizobium* | | 0.70 | | 5.64E-10 |
| *Streptococcus* | *Burkholderia* | | 0.69 | | 1.12E-09 |
| *Arthrobacter* | *Halomonas* | | 0.69 | | 1.55E-09 |
| *Gordonia* | *Tsukamurella* | | 0.68 | | 1.62E-09 |
| *Methylobacterium* | *Arthrobacter* | | 0.68 | | 2.53E-09 |
| *Arthrobacter* | *Undibacterium* | | 0.68 | | 2.59E-09 |
| *Streptococcus* | *Uruburuella* | | 0.67 | | 5.29E-09 |
| *Halomonas* | *Tsukamurella* | | 0.66 | | 1.10E-08 |
| *Enterococcus* | *Salinivibrio* | | 0.66 | | 1.13E-08 |
| *Brevundimonas* | *Rhizobium* | | 0.65 | | 1.68E-08 |
| *Pseudonocardia* | *Dietzia* | | 0.64 | | 2.84E-08 |
| *Gordonia* | *Halomonas* | | 0.64 | | 4.31E-08 |
| *Streptococcus* | *Salinivibrio* | | 0.63 | | 7.63E-08 |
| *Lactobacillus* | *Enhydrobacter* | | 0.63 | | 9.00E-08 |
| *Methylobacterium* | *Pseudonocardia* | | 0.62 | | 1.45E-07 |
| *Gordonia* | *Dietzia* | | 0.62 | | 1.61E-07 |
| *Arthrobacter* | *Dietzia* | | 0.60 | | 4.87E-07 |
| *Streptococcus* | *Enterococcus* | | 0.60 | | 5.38E-07 |
| *Undibacterium* | *Tsukamurella* | | 0.60 | | 5.43E-07 |
| *Arthrobacter* | *Ralstonia* | | -0.60 | | 8.13E-07 |
| *Burkholderia* | *Pelomonas* | | -0.60 | | 3.67E-07 |
| *Pelomonas* | *Salinivibrio* | | -0.64 | | 4.78E-08 |
| *Gordonia* | *Ralstonia* | | -0.65 | | 2.35E-08 |
| **Spearman correlation between genera in EC** | | | | | |
| **Source** | | **Target** | | **Spearman r value** | ***p*-value** |
| *Arthrobacter* | | *Tsukamurella* | | 0.83 | 1.52E-08 |
| *Burkholderia* | | *Uruburuella* | | 0.82 | 3.56E-08 |
| *Bacillus* | | *Uruburuella* | | 0.79 | 1.61E-07 |
| *Pseudonocardia* | | *Arthrobacter* | | 0.79 | 2.45E-07 |
| *Flavobacterium* | | *Cellvibrio* | | 0.72 | 6.55E-06 |
| *Bacillus* | | *Salinivibrio* | | 0.71 | 1.15E-05 |
| *Arthrobacter* | | *Gordonia* | | 0.70 | 2.00E-05 |
| *Brevundimonas* | | *Staphylococcus* | | 0.69 | 2.32E-05 |
| *Uruburuella* | | *Salinivibrio* | | 0.69 | 2.47E-05 |
| *Burkholderia* | | *Salinivibrio* | | 0.69 | 2.74E-05 |
| *Arthrobacter* | | *Bosea* | | 0.67 | 4.60E-05 |
| *Tsukamurella* | | *Bosea* | | 0.66 | 6.61E-05 |
| *Brevundimonas* | | *Enhydrobacter* | | 0.65 | 1.11E-04 |
| *Bacillus* | | *Burkholderia* | | 0.65 | 1.13E-04 |
| *Methylobacterium* | | *Arthrobacter* | | 0.65 | 1.15E-04 |
| *Sphingobium* | | *Anoxybacillus* | | 0.64 | 1.27E-04 |
| *Bacillus* | | *Streptococcus* | | 0.64 | 1.37E-04 |
| *Enterococcus* | | *Uruburuella* | | 0.64 | 1.48E-04 |
| *Novosphingobium* | | *Tepidimonas* | | 0.63 | 2.02E-04 |
| *Acinetobacter* | | *Pseudomonas* | | 0.60 | 4.73E-04 |
| *Tepidimonas* | | *Cloacibacterium* | | 0.60 | 4.99E-04 |
| *Pseudonocardia* | | *Tsukamurella* | | 0.60 | 5.06E-04 |
| *Sphingomonas* | | *Tsukamurella* | | 0.60 | 5.13E-04 |
| *Cellvibrio* | | *Salinivibrio* | | 0.60 | 5.29E-04 |
| *Anoxybacillus* | | *Pelomonas* | | -0.72 | 5.90E-06 |
| **Spearman correlation between genera in AC** | | | | | |
| **Source** | **Target** | | | **Spearman r value** | ***p*-value** |
| *Arthrobacter* | *Tsukamurella* | | | 0.84 | 5.46E-09 |
| *Arthrobacter* | *Gordonia* | | | 0.82 | 2.28E-08 |
| *Thermus* | *Brevibacillus* | | | 0.82 | 3.71E-08 |
| *Corynebacterium* | *Propionibacterium* | | | 0.81 | 7.50E-08 |
| *Pseudonocardia* | *Gordonia* | | | 0.71 | 1.06E-05 |
| *Pseudonocardia* | *Arthrobacter* | | | 0.70 | 1.57E-05 |
| *Gordonia* | *Tsukamurella* | | | 0.69 | 2.07E-05 |
| *Gordonia* | *Rhizobium* | | | 0.67 | 4.84E-05 |
| *Burkholderia* | *Salinivibrio* | | | 0.66 | 6.61E-05 |
| *Arthrobacter* | *Rhizobium* | | | 0.65 | 9.74E-05 |
| *Brevibacillus* | *Anoxybacillus* | | | 0.64 | 1.60E-04 |
| *Bacillus* | *Salinivibrio* | | | 0.63 | 1.95E-04 |
| *Thermus* | *Anoxybacillus* | | | 0.63 | 2.11E-04 |
| *Pseudomonas* | *Enhydrobacter* | | | 0.62 | 2.54E-04 |
| *Leptotrichia* | *Haemophilus* | | | 0.61 | 3.57E-04 |
| *Streptococcus* | *Delftia* | | | 0.60 | 4.11E-04 |
| *Pseudomonas* | *Massilia* | | | 0.60 | 4.23E-04 |
| *Burkholderia* | *Uruburuella* | | | 0.60 | 4.42E-04 |
| *Dietzia* | *Undibacterium* | | | 0.60 | 5.15E-04 |
| *Pseudonocardia* | *Haemophilus* | | | -0.63 | 2.17E-04 |
| *Arthrobacter* | *Helicobacter* | | | -0.64 | 1.63E-04 |
| *Anaerococcus* | *Undibacterium* | | | -0.66 | 8.30E-05 |
| *Nocardioides* | *Pelomonas* | | | -0.69 | 2.72E-05 |
