# Supplementary material for: Gastric Mucosa-Associated Microbial Signatures of Early Gastric Cancer
Source: Front Microbiol. 2020 Jul 7;11:1548. doi: 10.3389/fmicb.2020.01548 (PMC7358557; doi:10.3389/fmicb.2020.01548)
Supplement: Supplementary file 4 [file Table_4.DOCX]

**Supplementary Table S4 Allele frequency of SNPs associated with gastric cancer.**

| **Gene** | **SNPs** | **Genotypes** | **CG (%)**  **(n=60)** | **EC (%)**  **(n=30)** | **AC (%)**  **(n=30)** |
| --- | --- | --- | --- | --- | --- |
| *PSCA* | rs2920299 | AA | 19 (31.7) | 8 (26.7) | 12 (40.0) |
|  |  | AG | 31 (51.7) | 18 (60.0) | 13 (43.3) |
|  |  | GG | 10 (16.6) | 4 (13.3) | 5 (16.7) |
| *PSCA* | rs2976392 | GG | 38 (63.3) | 19 (63.3) | 17 (56.7) |
|  |  | GA | 20 (33.3) | 10 (33.3) | 11 (36.7) |
|  |  | AA | 2 (3.4) | 1 (3.4) | 2 (6.6) |
| *UNC5CL* | rs2294693 | TT | 36 (60.0) | 17 (56.7) | 14 (46.7) |
|  |  | TC | 22 (36.7) | 7 (23.3) | 14 (46.7) |
|  |  | CC | 2 (3.3) | 6 (20.0) | 2 (6.6) |
| *PRKAA1* | rs80315667 | DEL.DEL. | 33 (55.0) | 24 (80.0) | 18 (60.0) |
|  |  | DEL.T | 12 (20.0) | 4 (13.3) | 9 (30.0) |
|  |  | TT | 15 (25.0) | 2 (6.7) | 3 (10.0) |
| *PTGER* | rs10036575 | TT | 10 (16.7) | 11 (36.7) | 12 (40.0) |
|  |  | TC | 34 (56.6) | 13 (43.3) | 14 (46.7) |
|  |  | CC | 16 (26.7) | 6 (20.0) | 4 (13.3) |
| *MUC1* | rs4072037 | TT | 20 (66.7) | 47 (78.4) | 23 (76.7) |
|  |  | TC | 8 (26.6) | 11 (18.3) | 6 (20.0) |
|  |  | CC | 2 (6.7) | 2 (3.3) | 1 (3.3) |
