# Supplementary material for: Gastric Mucosa-Associated Microbial Signatures of Early Gastric Cancer
Source: Front Microbiol. 2020 Jul 7;11:1548. doi: 10.3389/fmicb.2020.01548 (PMC7358557; doi:10.3389/fmicb.2020.01548)

**Legends of Supplementary Figures**

**Supplementary Figure S1** Classification of different pathology using optimal RF model of 24 genera. ROC curves of the optimal model for classifying CG without intestinal metaplasia from EC (**A**), or with intestinal metaplasia from EC (**B**), and classifying CG from AC (**C**).

**Supplementary Figure S2** Validations of the identified microbial signature associated with EC in a Singapore cohort. ROC curve of the optimal model for classifying gastric cancer from functional dyspepsia.

**Supplementary Figure S3** Impacts of host SNPs on the gastric microbiome. Constrained PCoA were performed to explore the influence of SNPs on the gastric microbiome. These SNPs included rs2920299 and rs2976392 in *PSCA*, rs2294693 in *UNC5CL*, rs80315667 in *PRKAA1*, rs10036575 in *PTGER*, and rs4072037 in *MUC1* genes.

**Figure S1**


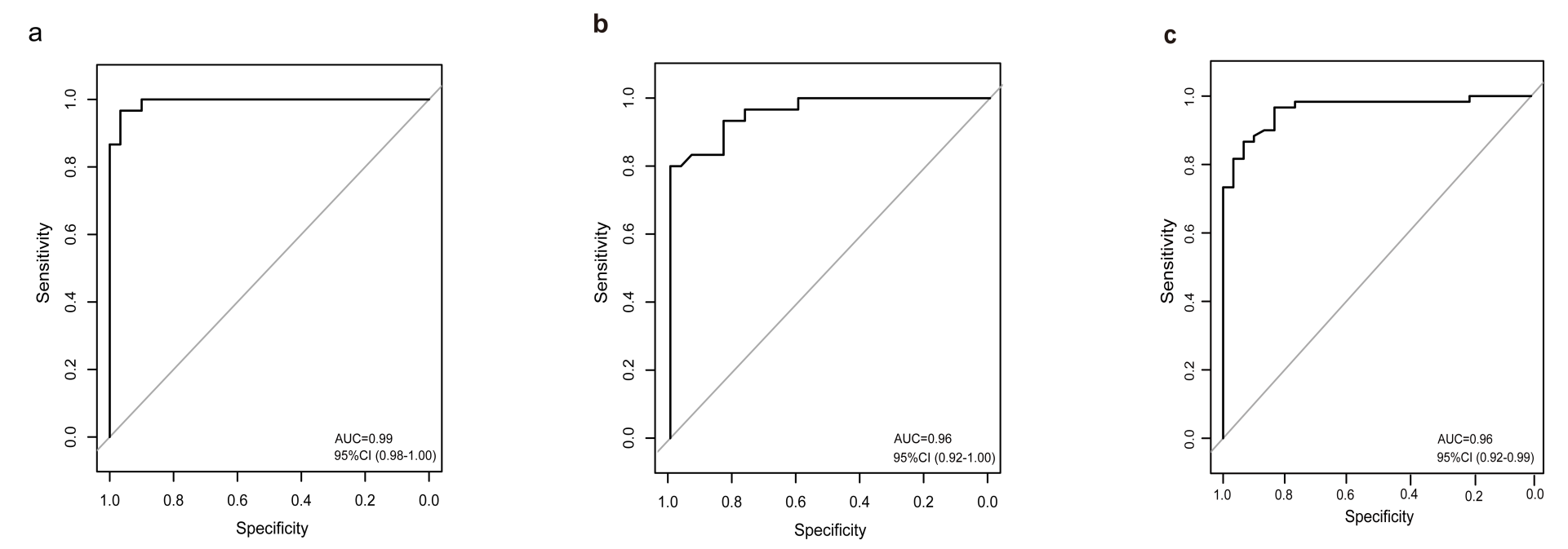


**Figure S2**


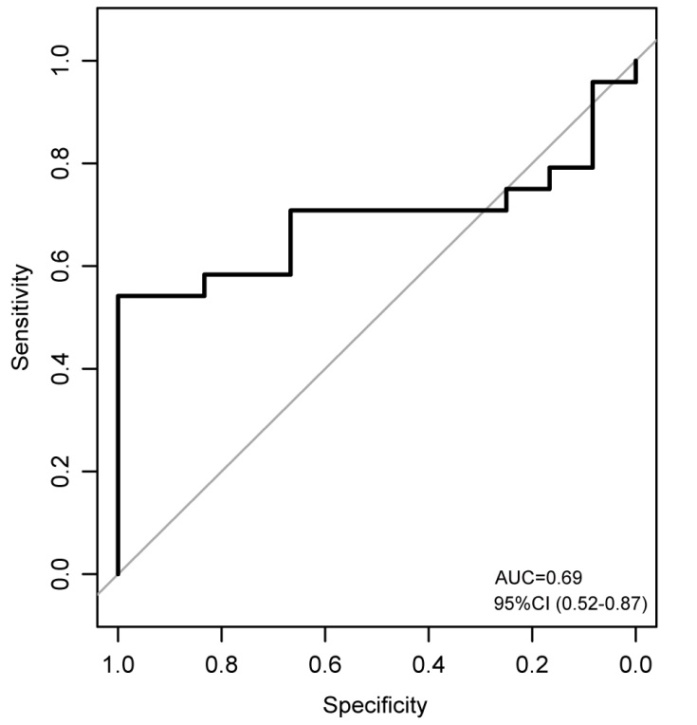


**Figure S3**

##
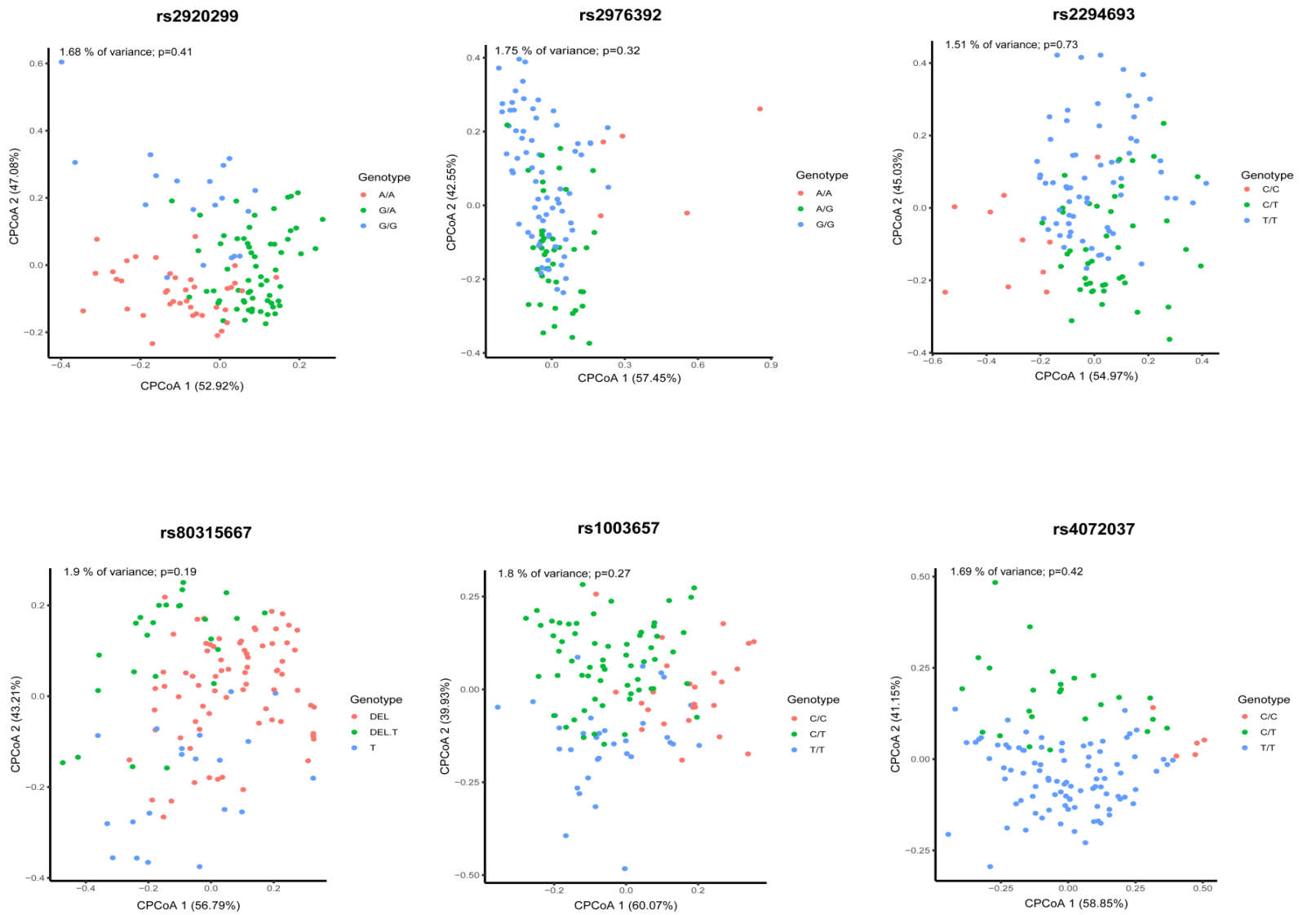

Supplement: Supplementary file 5 [file Data_Sheet_1.DOCX]
